# Psychometric Properties of the Posttraumatic Stress Disorder Checklist among the Lebanese Population Exposed to the Beirut Explosion: A Cross-Sectional Study during the COVID-19 Pandemic

**DOI:** 10.1155/2023/9286562

**Published:** 2023-10-03

**Authors:** Fadwa Alhalaiqa, Othman A. Alfuqaha, Rami Masa'Deh, Anas H. Khalifeh, Mahmoud Alsaraireh, Natija S. Manaa, Osama Alkouri, Omar Al Omari

**Affiliations:** ^1^Department of Clinical Affairs, College of Nursing, QU Health, Qatar University, Doha, Qatar; ^2^Counseling and Mental Health Department, Faculty of Educational Sciences, The World Islamic Sciences & Education University, Amman 11947, Jordan; ^3^School of Nursing, Applied Science Private University, Jordan; ^4^Department of Community and Mental Health Nursing, Faculty of Nursing, Zarqa University, P.O.Box 132222, Zarqa 13132, Jordan; ^5^Princess Aisha Bint Al Hussein College for Nursing and Health Sciences, Alhussein Bin Talal University, Ma'an, Jordan; ^6^Amman, Jordan; ^7^Faculty of Nursing, Yarmouk University, Irbid, Jordan; ^8^Faculty of Nursing, Sultan Qaboos University, Oman

## Abstract

**Objective:**

This study was aimed at testing the psychometric properties of the posttraumatic stress disorder (PTSD) checklist for the Diagnostic Statistical Manual version 5 (DSM-5) (PCL-5) among the Lebanese population and at identifying the prevalence of PTSD.

**Design:**

A cross-sectional survey of PCL-5 among 950 Lebanese, using the online survey platform by Google Form, was conducted. Snowball recruitment was used to identify participants for the survey.

**Results:**

Face, content, construct, discriminant, and convergent validity had been accomplished through the survey. The reliability using Cronbach's alpha, composite, and average variance extracted was identified as superior. We also found that more than half of the participants (55.6%) scored 33 or above which is the cut-off score for a likely diagnosis of PTSD.

**Conclusion:**

The current study provides further support for the validity and reliability of the Arabic version of PCL-5 among non-Western populations. This supports using the checklist in the screening of probable PTSD.

## 1. Introduction

Since December 2019, COVID-19 has had an impact on all elements of life: economic, social, educational, physical, and psychological. Several studies have found that during the COVID-19 pandemic, there was a surge in mental health problems in the Arabic region [1–5]. Stress, panic, anxiety, depression, obsessive-compulsive disorders, and posttraumatic stress disorder (PTSD) were among the issues that have been identified [6] and were especially prevalent among refugees and displaced individuals [6]. Furthermore, an explosion in Beirut's port in August 2020 resulted in the spill of nearly 2,750 tons of ammonium nitrate. As a result of this, more than 200 people were killed, 6,500 were injured, and over 300,000 people were forced to abandon their homes [7, 8]. This event exacerbated the economic, social, and psychological impact of the COVID-19 pandemic and its associated lockdowns [7]. PTSD is one psychological problem that has had a clear appearance and increase with the presence of COVID-19 worldwide with prevalence estimates ranging from 7.0% to 72% [9–15]. In the Arab region, the prevalence was high even with the use of different scales to measure it; in a study conducted in seven Arab countries to investigate the impact of COVID-19 on mental health, 36.6% of participants were diagnosed with probable PTSD [16]. Ibrahim et al. [13] investigated adult Egyptians who survived the COVID-19 virus infection and found that around 72% experienced moderate to severe posttraumatic stress symptoms using the Impact of Event Scale-Revised (IES-R). In Saudi Arabia, three months following the initial COVID-19 quarantine, a study of 1,374 Saudi individuals indicated a 19.6% prevalence of PTSD [15]. The prevalence of posttraumatic stress symptomology (PTSS) among 950 civilians was evaluated because of the COVID-19 quarantine on two time series ranging from two weeks to one month; they used the PTSD Checklist–Civilian Version (PCL-C) [9]. Their findings revealed that PTSS was more prevalent in the fourth week compared to the second week (33.2% in the second week vs. 62.63% in the fourth week) [9].

PTSD usually occur after traumatic occurrences that exceed the range of common human experience (for example, domestic violence, rape, torture, warfare, accidents, human-made, and natural disasters) that affect the mind and behavior of the individual [10, 11]. It is characterized by a typical symptom pattern of disturbing thought process, emotional numbness, attempts to avoid trauma-related cues, and physiological hyper-arousal [10].

The most reported studies used self-reported measures for the assessment of PTSD, particularly the PTSD Checklist for DSM-5 (PCL-5) which consists of 20 items [9]. Therefore, in most cases, it is just symptomatology that has been observed and not an overt disorder. Since all measures only assess PTSS, only a diagnosis of probable PTSD can be made for those who exceed the suggested cut-off for each measure.

The COVID-19 emergency's specific qualities could lead to a new perspective on trauma, which may differ from these previously defined PTSD criteria. The Beirut explosion might be worsening the traumatic experience of the Lebanese COVID-19 pandemic. Therefore, the current study was aimed at evaluating the psychometric properties of PCL-5 in the context of the COVID-19 epidemic and at determining the PTSD prevalence in a sample of Lebanese populations after the Beirut explosion.

## 2. Materials and Methods

A cross-sectional survey was used to investigate the current study's aims using an online survey. The data was collected from November 2020 to April 2021. The STROBE guideline was used in reporting the current study.

### 2.1. Participants and Procedure

To collect the data, we used an online survey hosted by Google Drive. The snowball procedure was selected to distribute the survey. This generates a pool of participants who had contact with initial participants through social network websites. Very large samples are essential to test the psychometric properties of assessment scales. In 2014, a review of 114 relevant articles found that 90% of articles had a sample size greater than 100 participants and only 7% had greater than or equal 1,000 participants [12]. The sample size was calculated using the G-power equation with a relatively small effect size (0.15), 80% of statistical power, and significance level of 0.05; the estimated sample size is 492 participants. Increasing the sample size would increase the study's power and confidence level. Additionally, we expected to have an incomplete questionnaire; therefore, we decided to expand the period of data collection to achieve the maximum sample size. A total of 891 participants were enrolled in this study. The inclusion criteria were people above 18 years old, living in Lebanon, exposed to the Beirut explosion, and who experienced the period of COVID-19 outbreaks.

### 2.2. Research Ethics Approval

Ethical approval was obtained from the institutional review board (IRB) of the primary investigator's university. The Declaration of Helsinki was followed in the current study. The online survey started with an explanation of the purpose of the study and assured participants of their rights (e.g., to withdraw or to decline to participate, confidentiality, and anonymity). Potential participants were asked to explicitly consent to take part in the study and had to press “yes” before being transferred to fill the scale.

### 2.3. Patient and Public Involvement

The participants were involved in developing research questions and designing and conducting this research. Before conducting the study, a pilot study was done. The online translated Arabic version of the instrument was sent by WhatsApp to the focus group (group of five persons from Beirut/Lebanon) who filled the questionnaires. They were also asked to give their feedback about the readability and understandability of the instrument. Then, an online discussion was conducted with this focus group, and a slight modification was made with maintaining the conceptual meaning of the items. Additionally, an interview was conducted with two experts from Lebanon (one was a psychiatrist, and the other was a social worker) who approved the preliminary face validity. However, we did not include the pilot's study participants in the analysis. The participants themselves helped in the recruitment of expected participants and in conducting the study, since our sampling method was a snowball technique. Once the paper has been published, it will be disseminated through the PI's social media, and the findings will be sent to a suitable newsletter to be published for a nonspecialist audience.

### 2.4. The Instrument and Translation

The survey had two parts. Part one collected information on demographics (e.g., age, gender, and educational level), clinical characteristics (e.g., visits to psychiatric units, loss of friends/family, being infected with COVID-19, and comorbidities) (how the participants experienced the COVID-19 outbreak was measured by answering the question, “have you been infected with COVID-19”), and lifestyle (e.g., patterns of sleep, smoking habits, work characteristics, and family characteristics).

The second part of the questionnaire was the PTSD Checklist for DSM*-5* (PCL-5). It is a 20-item questionnaire which assesses symptoms such as “repeated, disturbing dreams of the stressful experience” with a Likert scale ranging from 0 (not at all) to 4 (extremely). It has four dimensions: intrusion, emotion alteration, avoidance, and hyper-arousal. A total score is computed from summating each item's score, with higher scores indicating a higher degree of PTSD symptoms. It has good validity and reliability within different languages (Arabic, Kurdistan, and English versions) [9, 13, 14]. It had strong internal consistency (*α* = .95) and test–retest reliability (*r* = .90) [9, 14]. However, no study was found that investigated the complete psychometric proprieties of the Arabic version. We used the same cut-off point that has been used by Alshehri et al. [15] and Ahmad et al. [16] in their studies (among Saudi, Kurdish, and Arab populations, respectively).

The translation process of the original scale was examined by four bilingual independent experts specialized in English-Arabic translation, nursing, and psychology. We used both forward and backward translations. We used, in the first step, forward translations from the English language to Arabic language taking into consideration the original term and translating it to the relevant term, using simple, clear, and fewer words that are the cultural equivalent [17]. The second step was backward translations. In this regard, the selected independent experts did the reverse translations into the English language. The translators agreed on the final Arabic version of PCL-5. The independent experts agreed on the combined version translations, and they reached 100% agreement on the translation of the 20 items of PCL-5.

### 2.5. Analysis Plan

The analysis process for the translated PCL-5 encompassed various facets of validity, including face validity, content validity, and construct validity, as well as measures of convergent and discriminant validity. Construct validity was assessed through exploratory factor analysis (EFA) with varimax rotation, focusing on factor loadings exceeding 0.40 and eigenvalues greater than 1. Convergent and discriminant validity was assessed through analyzing bivariate correlations between constructs. It ensured that these correlations did not exceed the average variance extracted (AVE) for each construct. The overall AVE surpassed the 0.50 threshold, validating the comprehensive representation of each construct within the scale [18]. To assess the factor structure of the translated PCL-5, confirmatory factor analysis (CFA) was employed. The validation criteria used for evaluating the model fit were as follows: relative chi-square (*χ*^2^/df ratio > 3), root mean square of error approximation (RMSEA < 0.05), comparative fit index (CFI > 0.90), goodness-of-fit index (GFI > 0.90), increment fit index (IFI > 0.90), and Tucker-Lewis index (TLI > 0.90), and modification indices above 15 indicate a redundancy model [19, 20]. To assess the reliability of the translated PCL-5, composite reliability (CR), AVE, and Cronbach's alpha were detected. The values greater than or equal to 0.70 are considered consistent in CR [21]. Cronbach's alpha values greater than or equal 0.80 are preferred [22]. This process was done in several articles such as by Alfuqaha et al. [23].

## 3. Results

### 3.1. Demographic Characteristics

A total of 891 individuals participated in this study. The characteristics of the participants are presented in Table 1. Most of the selected sample were female, held postgraduate degrees, experienced sleep issues, but had not sought treatment in psychiatric units. The average age of the participants was 35.4 years. Notably, 36.8% of respondents reported the loss of at least one friend due to the COVID-19 pandemic. Additionally, nearly one-third of the sample had contracted COVID-19 and had comorbid health conditions.

### 3.2. Validity and Reliability of PCL-5

To validate the PCL-5 in the Arabic language, we followed the steps outlined as follows:

#### 3.2.1. Face Validity

To assess the quantitative face validity of the PCL-5, a pilot study involving 30 participants from the Lebanese population was conducted. Participants were asked to provide feedback and suggestions concerning various aspects, including context, linguistic accuracy, ambiguity, local relevance, and simplicity of the translated version of the PCL-5. We constructed a table to record the importance scores, which were rated on a 3-point Likert-type scale including “very important,” “important,” and “not important.” An important score of equal to or greater than 1.5, as indicated by the respondents, signified that the translated PCL-5 had clear and understandable items [24]. None of the participants expressed concerns regarding incorrect or unclear items. All items met the criteria of 1.5 or higher, and consequently, none of the items were removed from the scale.

#### 3.2.2. Content Validity

The content validity ratio (CVR) was computed by presenting the PCL-5 to six academic experts spanning various fields, including medicine, humanities, and psychology. These experts were asked to evaluate the clarity of the items and indicate their agreement or disagreement. According to Lawshe's content validity ratio table, a minimum of 0.83 approval was required from the six experts. Upon collecting their responses, the CVI results showed an average score of 0.91. Therefore, no further items were deemed necessary to be removed from the PCL-5.

#### 3.2.3. Construct Validity

**Figure 1:**
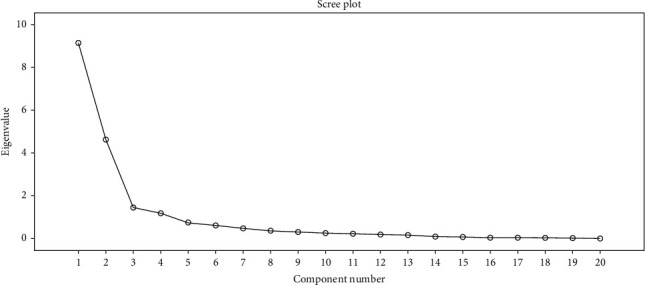
Scree plot for the translated PTSD checklist (20 items).

After running factor analysis, the result revealed in Table 2 that the translated PCL-5 exhibited four factor-loading models that collectively accounted for 81.9% of the variances. These models encompassed distinct constructs which are intrusive symptoms (6 items) with factor loadings ranging from 0.78 to 0.92, explaining 45.67% of the item variance with eigenvalues of 9.13. The second construct is avoidance of thoughts (7 items) with factor loadings between 0.55 and 0.90, explaining 23.14% of the item variance and eigenvalues of 4.63. The third construct is negative alterations to mood (5 items) with factor loadings ranging from 0.55 to 0.89, explaining 7.15% of the item variance and eigenvalues of 1.43. The fourth construct is alterations in lifestyle (2 items) with factor loadings of 0.84 and 0.66, explaining 5.92% of the item variance with eigenvalues of 1.18. The scree plot corroborated these results (Figure 1), demonstrating significant correlations among the subscales themselves and with the total score of the PCL-5. The results indicated that the translated PCL-5 attained sampling adequacy with superior data quality.

#### 3.2.4. Convergent and Discriminant Validity

An assessment of convergent validity was conducted by computing both CR and AVE. Consistent with the literature, it is essential that CR values for all constructs within the PCI-5 exceed the threshold of 0.70. Furthermore, it is imperative that the AVE values surpass the threshold of 0.50. As depicted in Table 3, all the obtained results align with the established standard values. To assess discriminant validity, we examined the intercorrelations between the exogenous constructs of the translated PCL-5 and compared them with the square root of the AVE. The presence of discriminant validity is supported when the interconstruct correlations are lower than the square root of the AVE values, as demonstrated in Table 3.

### 3.3. Confirmatory Factor Analysis

**Figure 2:**
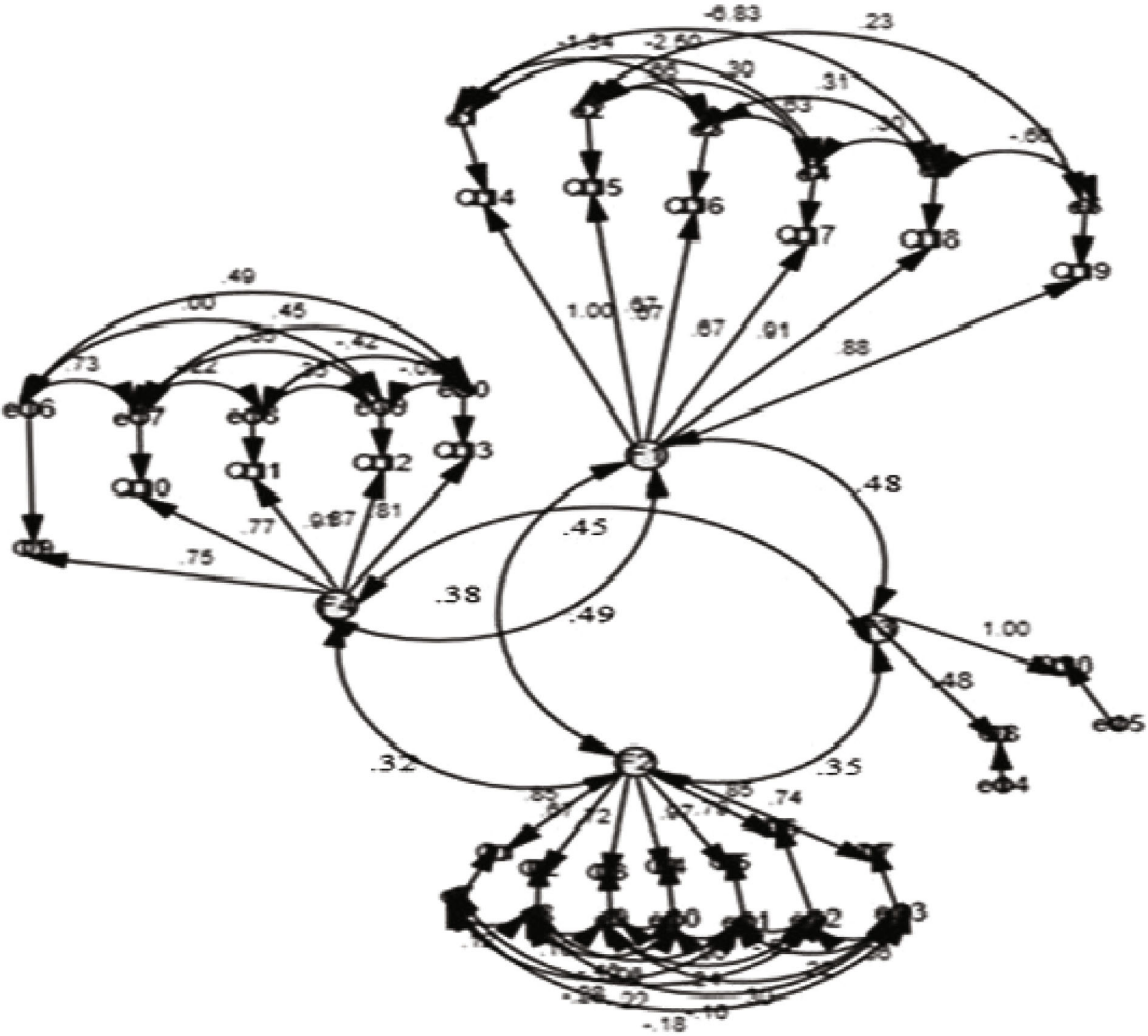
CFA measurement model 2 for the translated PCL-5 (AMOS V26). F1: intrusive symptoms; F2: avoidance of thoughts; F3: alterations in lifestyle; F4: negative alterations to mood.

The CFA was employed to assess the adequacy of the latent construct models of the translated PCL-5 in relation to the available data. In the initial model run, the required fit level, as indicated by standard fitness indices, was not met, and redundancy was observed. Consequently, we conducted free parameter estimates, as illustrated in Figure 2. Subsequently, we reran the model, resulting in the following fit indices: *χ*^2^/df: 7455.3/130, RMSEA: 0.04, CFI: 0.96, GFI: 0.95, IFI: 0.96, and TLI: 0.94. These findings demonstrate that the model now satisfies the criteria for proper fitness indices.

## 4. Reliability

Cronbach's alpha, CR, and AVE collectively affirm the internal reliability of the translated scale. As presented in Table 3, all constructs have attained the necessary thresholds, further validating the scale's internal consistency.

### 4.1. The Level of PTSD among Participants and Its Demographic Correlation

The cut-off point scale for PTSD is 33. A total of 495 participants (55.6%) had achieved the criteria of PTSD with a scale mean (SD) score of 40.7 (15.9) (see Table 4).

## 5. Discussion

The current study found that the translation of PCL-5 to the Arabic language was achieved by experts in the related field. Face, content, construct, discriminant, and convergent validity had been proven. Furthermore, the reliability using Cronbach's alpha, composite, and average variance extracted was identified as superior. Additionally, the findings revealed that the prevalence of PTSD among Lebanese of the post-Beirut explosion was 55.6 using a translated Arabic version of PCL-5. These findings to some degrees were consistent with French version psychometric properties which demonstrated excellent internal consistency (*α* = .94) and strong convergent and divergent validity [25].

A study was conducted among 950 Lebanese at two time intervals (2-4 weeks) during the COVID-19 quarantine to measure the prevalence of posttraumatic stress symptomatology (PTSS). Their results showed that the PTSS started to rise at 2 weeks and worsen at 4 weeks [26]. In the current study, we measured the PTSD after around 2 years of COVID-19 and 4-9 months after the Beirut explosion. We found that more than half of the participants (55.6%) scored 33 or above which is the cut-off score for likely PTSD diagnosis. This prevalence is considered high in comparison to previous studies conducted during COVID-19; for example, in Turkey, it was 33.2% [27]; in China, it was 13.2% among hospitalized individuals [28]; and in Norway, it was 9.5% in hospitalized and 7% in nonhospitalized adults [29].

The prevalence of PTSD might be aggravated by the Beirut explosion. However, other characteristics of this population could explain the high prevalence of PTSD; for example, most of the current study's participants were female, have sleep problems [28, 29], and were displaced people [17, 28].

The internal consistencies of the PCL-5 scores among Kurdish and Arabic Iraqi before COVID-19 were good with *α* = .85 and *α* = .86, respectively. Meanwhile, for PCL-5's subscales (intrusion, avoidance, negative alterations in cognition and mood, and alterations in lifestyle (hyper-arousal)) were *α* = .76, *α* = .88, *α* = .74, and *α* = .71, respectively [7]. However, in the current study, Cronbach's *α* coefficients for PCL-5 and its subscales were excellent (0.94, 0.93, 0.93, and 0.92), except for hyper-arousal symptoms in which Cronbach's *α* was 0.62. This finding was similar to the internal consistency of the French and Italian PCL-5 versions (*α* > .79 and >.95, respectively) [25, 30]. Additionally, current findings showed that the confirmatory factor analysis of the Arabic version of PCL-5 indicated a good factor structure. These findings are similar to the previous study which has been conducted among 375 Saudis before COVID-19 [17]. The convergent and discriminant validity of the Arabic version of PCL-5 in the current study is supported by the English version [27, 28, 31]. This indicated that the PCL-5 has excellent psychometric properties which could be used for the screening of PTSD. The PTSD checklist is a self-reported instrument to assess the symptoms and can help in the screening process. To confirm the diagnosis of PTSD, referral to a physician is needed.

The strengths of the current study were as follows: it is considered the first structural equation modeling (SEM) of the PCL-5 factor structure among the Lebanese people after the Beirut explosion which proved the existence of a relationship between the 20 items of the instrument and its constructs (the PCL-5 Arabic version has good psychometric properties) and used a good sample size in comparison with previous studies, in addition to having different nationalities (Lebanese, Syrian, and Palestinian) and a variety of religious backgrounds (Muslims and Christians). However, we did not measure nationality or religion. These must be considered in future research.

The current study is limited to using a cross-sectional design and snowball sampling technique. Additionally, we did not measure if the participants had any loss in terms of people or property as a result of the Beirut explosion. Additionally, the current study included people not only from Beirut (where the explosion occurred) but from all of Lebanon. All these limitations must be implemented in future research. We did not use other instruments to assess PTSD to analyze the relationship between different variables in the construct so that the psychometric properties of the PCL-5 could only be partially examined. This must be investigated by future research.

## 6. Conclusion

We conclude that the Arabic version of PCL-5 could be used to assess and screen for the symptoms of PTSD; the level of probable PTSD symptoms in the Lebanese community after the Beirut explosion was high. The current study provides further support for the validity and reliability of the Arabic version of PCL-5 among non-Western populations and the help it provides in screening PTSD symptomatology. The pandemic and Beirut explosion have had a negative impact on psychiatric mental health problems amongst the Lebanese. The findings of the current study highlight the need for urgent public interventions directed to manage PTSD symptoms. Government, policy makers, and nongovernmental organizations (NGOs) must design appropriate policies, allocate required personnel and resources, and build a management center that focus on psychological and mental health problems, particularly PTSD, to reduce the negative psychosocial, economic, and health problems.

We suggest that the Arabic version of PCL-5 could be used to screen for PTSD symptomatology, by community health care providers, the ministry of health, and psychiatric care providers. So, the referral for a psychiatric specialist for further diagnosis could be done. Increased community awareness and education about PTSD detection and management is needed to reduce this burden from the pandemic and other stressor events (e.g., the Beirut explosion). Future research is needed to determine the validity and reliability of PCL-5 among children.

## Figures and Tables

**Table 1:** Demographic characteristics of the study sample and association of demographic factors with translated PTSD scale (no. = 891).

Variable	Description	Frequency	Percentage (%)	*M* ± SD	*F*/*t*-test	*P* value
Gender	Male	216	24.2	0.80 ± 0.89	4.99	<0.01^∗∗^
	Female	675	75.8	1.11 ± 0.75		

Educational level	High school	27	3	0.80 ± 0.0		<0.01^∗∗^
	Diploma	279	31.3	1.50 ± 0.75	55.11	
	Bachelor	144	16.2	0.79 ± 1.01		
	Postgraduate	441	49.5	0.83 ± 0.62		

Comorbidities	Yes	315	35.4	1.36 ± 0.87	9.55	<0.01^∗∗^
	No	576	64.6	0.85 ± 0.68		

Visited psychiatric clinic	Yes	189	21.2	0.89 ± 0.74	332.50	<0.01^∗∗^
	No	558	62.6	0.78 ± 0.58		
	I need to	144	16.2	2.21 ± 0.47		

Lost one of your family due to COVID-19	Yes	63	7.1	0.91 ± 0.77	1.22	0.22
	No	828	92.9	1.04 ± 0.79		

Lost one of your friends due to COVID-19	Yes	328	36.8	0.91 ± 0.68	3.50	<0.01^∗∗^
	No	563	63.2	1.10 ± 0.84		

Lost job due to COVID-19	Yes	90	10.1	1.49 ± 0.56	5.87	<0.01^∗∗^
	No	801	89.9	0.98 ± 0.80		

Being infected with COVID-19	Yes	324	36.4	1.02 ± 0.71	0.21	0.89
	No	567	63.6	1.03 ± 0.84		

Sleep problems	Yes	531	59.6	1.26 ± 0.80	11.07	<0.01^∗∗^
	No	360	40.4	0.70 ± 0.65		

Smoking	Yes	351	39.4	1.41 ± 0.81	12.33	<0.01^∗∗^
	No	540	60.6	0.79 ± 0.68		

Working	Yes	424	47.6	0.75 ± 0.71	5.87	<0.01^∗∗^
	No	467	52.4	1.05 ± 0.60		

Family member				3.75 ± 2.62	—	—

Age (year)				35.41 ± 65.73	—	—

*M* ± SD: mean and standard deviation; *F*: *F*-distribution. ^∗∗^*P* value ≤ 0.01.

**Table 2:** Component analysis and eigenvalues for translated PCL-5.

PTSD scale	Factor 1	Factor 2	Factor 3	Factor 4
Item 1		0.66		
Item 2		0.55		
Item 3		0.83		
Item 4		0.80		
Item 5		0.81		
Item 6		0.73		
Item 7		0.90		
Item 8				0.84
Item 9			0.89	
Item 10			0.77	
Item 11			0.55	
Item 12			0.60	
Item 13			0.72	
Item 14	0.78			
Item 15	0.86			
Item 16	0.92			
Item 17	0.85			
Item 18	0.83			
Item 19	0.81			
Item 20				0.66
Initial eigenvalues	9.13	4.63	1.43	1.18
Percentages of variance explained	45.67	23.14	7.15	5.92
Cumulative variance	45.67	68.80	75.95	81.87

Factor 1: intrusive symptoms; factor 2: avoidance of thoughts; factor 3: negative alterations to mood; factor 4: alterations in lifestyle.

**Table 3:** Cronbach's alpha, CR, AVE, and bivariate correlations among constructs.

#	Constructs	1	2	3	4	Alpha	CR	AVE
1	Intrusive symptoms	0.92				0.93	0.94	0.85
2	Avoidance of thoughts	0.38	0.90			0.93	0.90	0.81
3	Negative alterations to mood	0.49	0.32	0.71		0.92	0.84	0.51
4	Alterations in lifestyle	0.48	0.35	0.45	0.67	0.62	0.72	0.58
	Total PTSD scale					0.94	0.97	1.34

CR: composite reliability; AVE: average variance extracted. Note: the diagonal line presents the squared root of AVE.

**Table 4:** Means, standard deviations, and overall level of the translated PCL-5.

	Mean	SD	Level
Intrusive symptoms			
Item 1	1.16	0.92	Mild
Item 2	0.48	0.84	Mild
Item 3	0.55	0.78	Mild
Item 4	1.14	1.10	Mild
Item 5	0.70	1.04	Mild
Item 6	0.86	1.09	Mild
Item 7	0.86	1.21	Mild
Total score of intrusive symptoms	0.82 (20.5%)	0.84	Mild
Alterations in lifestyle			
Item 8	0.73	0.99	Mild
Item 20	1.86	1.42	Moderate
Total score of alterations in lifestyle	1.20 (30%)	1.01	Mild
Negative alterations to mood			
Item 9	0.86	1.41	Mild
Item 10	0.70	1.10	Mild
Item 11	1.34	1.50	Moderate
Item 12	1.60	1.43	Moderate
Item 13	1.52	1.33	Moderate
Total score of negative alterations to mood	1.20 (30%)	1.19	Mild
Avoidance of thoughts			
Item 14	1.33	1.48	Mild
Item 15	1.02	1.29	Mild
Item 16	0.61	1.15	Mild
Item 17	1.17	1.14	Mild
Item 18	0.85	1.14	Mild
Item 19	1.51	1.34	Moderate
Total score of avoidance of thoughts	1.08 (27%)	1.08	Mild
Total score of PTSD	1.03 (25.75%)	0.80	Mild

## Data Availability

The data that support the findings of this study are available on request from the corresponding author (FA). The data are not publicly available due to their containing information that could compromise the privacy of research participants.
